# Unraveling Mitochondrial Determinants of Tumor Response to Radiation Therapy

**DOI:** 10.3390/ijms231911343

**Published:** 2022-09-26

**Authors:** Mattia Zaffaroni, Maria Giulia Vincini, Giulia Corrao, Giulia Marvaso, Matteo Pepa, Giuseppe Viglietto, Nicola Amodio, Barbara Alicja Jereczek-Fossa

**Affiliations:** 1 Division of Radiation Oncology, IEO—European Institute of Oncology, IRCCS, 20141 Milan, Italy; 2 Department of Oncology and Hemato-Oncology, University of Milan, 20122 Milan, Italy; 3 Department of Experimental and Clinical Medicine, Magna Graecia University of Catanzaro, 88100 Catanzaro, Italy

**Keywords:** mitochondria, radiotherapy, radioresistance, ROS, tumor hypoxia, mitochondria-targeting compounds

## Abstract

Radiotherapy represents a highly targeted and efficient treatment choice in many cancer types, both with curative and palliative intents. Nevertheless, radioresistance, consisting in the adaptive response of the tumor to radiation-induced damage, represents a major clinical problem. A growing body of the literature suggests that mechanisms related to mitochondrial changes and metabolic remodeling might play a major role in radioresistance development. In this work, the main contributors to the acquired cellular radioresistance and their relation with mitochondrial changes in terms of reactive oxygen species, hypoxia, and epigenetic alterations have been discussed. We focused on recent findings pointing to a major role of mitochondria in response to radiotherapy, along with their implication in the mechanisms underlying radioresistance and radiosensitivity, and briefly summarized some of the recently proposed mitochondria-targeting strategies to overcome the radioresistant phenotype in cancer.

## 1. Introduction

It is historically known that mitochondria developed from an endosymbiotic association between an ancestral bacteria and a proto-eukaryotic host cell, inaugurating a two billion-year symbiotic partnership [1]. Since then, it has been recognized that the mitochondrion is a highly evolved system coordinating energy production and distribution for cellular maintenance and reproduction. Specifically, mitochondria are small, double-membrane organelles whose main task is to ensure general cellular metabolism and the supply of energy by means of ATP through the tricarboxylic acid cycle, electron transport chain (ETC), and oxidative phosphorylation (OXPHOS). While their main function is to serve as metabolic units, mitochondria have also co-evolved with their hosts to function as central signaling nodes in multiple pathways, involved in modulation of the intracellular redox status, reactive oxygen species (ROS) production, and modulation of inflammation and apoptosis. Over the years, mitochondrial DNA (mtDNA) has evolved alongside its nuclear counterpart (nDNA); however, given the relatively small size of the mitochondrial genome, consisting of 37 genes, it has often been ignored in the pioneering sequencing analyses and functional studies. Nevertheless, an increasing number of studies is reporting the influence of mutations in the mtDNA sequence in a large variety of diseases, from metabolic and musculoskeletal ones to cancer [2,3,4].

Overall, mitochondria may contribute to malignant transformation by two major mechanisms, also discussed in more specialized reviews: (i) ROS production, which potentially contributes to the accumulation of DNA mutations and to the activation of oncogenic pathways [5]; and (ii) mitochondria outer membrane permeability transition which is a crucial step required for malignant clones to escape programmed cell death [6,7]. In addition, a third mechanism has recently emerged, consisting in the abnormal accumulation of mitochondrial metabolites (i.e., fumarate and succinate), resulting in transforming effects from normal to malignant clones [8].

In many tumors, radiotherapy (RT) represents the first line of treatment, both with curative and palliative intent, and it is estimated that as many as half of all cancer patients will receive RT at some point throughout the course of disease [9]. Growing evidence suggests that specific mitochondrial changes and metabolic remodeling play a role in the onset of resistance to RT [10,11,12,13]. Radioresistance, defined as the adaptation of tumor cells to ionizing radiation (IR)-induced damages, represents a major clinical issue in various cancer types [14,15]. Its etiology is complex and includes interactions among various cellular mechanisms, such as DNA damage repair mechanisms, cell cycle arrest, oncogenes and/or tumor suppressor genes, tumor microenvironment (TME), microbiome changes, and altered regulation of ROS [12,16].

The present review highlights recent findings on the role of mitochondria in RT, focusing on their implication in the mechanisms underlying radioresistance and radiosensitivity and on the available mitochondria-targeting strategies in the radioresistant setting. A brief focus will be also given to the cross-talk among hypoxia-induced radioresistance, ROS regulation, metabolic reprogramming, and epigenetics.

## 2. Radiation Therapy and Mitochondria

The long-held concept of radiation biology assumed that the effects of IRs were a direct result of targeted DNA damage in the nuclei of impacted cells. On the other hand, non-targeted effects and acquired genomic instability suggested that such a model could be flawed [17,18]. A significant body of the literature reported that IR exposure results in a long-term increase in oxidative stress [19]. In particular, cumulative targeting of mitochondrial metabolism and that of several redox-sensitive pathways by radiation were proven fundamental in elevating oxidative injury and altering cellular physiology within the intracellular microenvironment. The current hypothesis is that oxidative stress might promote an unstable phenotype functioning as a hypothetically unifying biochemical framework that might link multiple seemingly disparate cellular responses to past radiation assaults [20,21,22].

**Figure 1:**
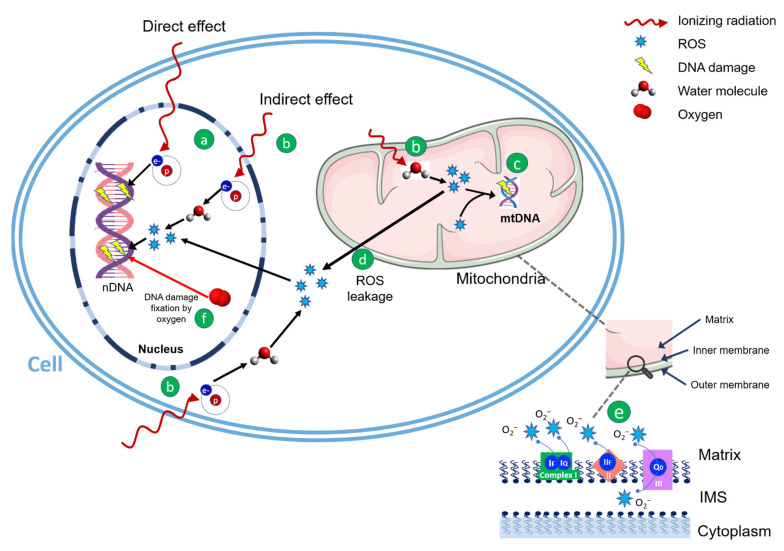
DNA damage by IR can be direct or ROS-mediated. In the direct effect (a), DNA molecules are hit directly by the secondary electrons produced by the incident radiation, resulting in cleavage of the chemical bonds and lesions such as single- and double-strand breaks. In the indirect effect (b), secondary electrons interact with water to produce ROS which attack DNA molecules in the cell, in the nucleus and in the mitochondria. When mitochondria are exposed to IR, the generation of ROS rises and can harm mtDNA in the matrix (c) and nDNA by leakage in the cell (d). Some of the recognized locations for ROS formation during oxidative phosphorylation in ETC are shown in the bottom right (e) and include complexes I and III, which are the primary sources of ROS in mitochondria, as well as complex II. The most prevalent ROS in mitochondria are superoxide anions, extremely reactive free radicals that are easily changed into other ROS such as hydrogen peroxide (H_2_O_2_) and hydroxyl ions (OH). The indirect ROS-mediated effect of IR is enhanced in the presence of oxygen: under aerobic conditions, oxygen reacts extremely rapidly with DNA radicals, fixating the damage and ensuring an unrepairable strand break (f); in the absence of oxygen, DNA radicals can be reduced, and DNA repairs to its original form, preventing strand damage. Abbreviations: ETC, electron transport chain; IMS, intermembrane space; IR, ionizing radiation; mtDNA, mitochondrial DNA; nDNA, nuclear DNA; ROS, reactive oxygen species.

RT is a critical component of many cancer treatments, and generally, its mechanism of action relies on the direct induction of DNA damage or the indirect production of ROS (Figure 1).

In the direct action, electrons directly interact with DNA, causing double- and single-strand breaks (DSBs and SSBs, respectively), ultimately leading to cell death. This process is predominant with dense IR, such as charged particles with enough kinetic energy and high-linear energy transfer (LET) radiations [23], as protons in the end region of their range. In the indirect action, predominantly within sparse IR (photons), water molecules, representing the major constituent of the cell (about 80%), are hit-producing free radicals (such as the hydrogen radical –H•– and hydroxyl radical –OH•–), which are very reactive molecules because of the unpaired orbital electron in their structures. In turn, this event triggers a chain reaction: free radicals are able to react with oxygen, fixating the radiation-induced modification and resulting in irreversible DNA damage [24,25]. This phenomenon is largely explained by the oxygen enhancement ratio (OER) or oxygen enhancement effect, which in radiobiology refers to the boosting of therapeutic or detrimental effects of IR due to the presence of oxygen. Since sparse ionizing radiation induces about 70% of the damage by indirect effect, oxygen is necessary for the fixation of DNA damage by ROS and the main consequence is that tumor hypoxia represents one of the most important factors in the development of radioresistance.

Mitochondria, being the hub of energy generation, are a key supplier of reactive species, especially when metabolic stress disrupts oxidative phosphorylation processes [26]. Depending on the cell type, they may constitute about 4% to 25% of the cell volume, therefore representing a sizeable target for IR [27].

In mammalian cells, under physiological conditions, mitochondria represent the most important source of ROS [28], with ATP synthesis producing them during normal oxygen metabolism and accounting for about the great majority (more than 90%) of the total cellular ROS generation [29,30]. As summarized in Figure 1, ROS are produced, when oxygen is reduced during aerobic respiration, spotted from complexes I, II, and III [31], within the ETC in the inner mitochondrial membrane (IMM), or by oxidoreductase enzymes and metal-catalyzed oxidation throughout the lifetime of the cell cycle [32]. As a result, about 5% of the oxygen consumed by mitochondria gives rise to ROS, ultimately leading to oxidative stress affecting both mt- and nDNA alongside with all the other cellular constituents [33,34].

In different malignancies, mitochondria enable a higher proliferation rate of cancer clones by increasing their energy metabolism through multiple mechanisms, including the switch to glycolysis (instead of OXPHOS) for ATP production [35,36] as well as other metabolic changes, conferring both the ability to evade apoptosis as well as protection from chemical- or radiation-induced damages [37].

Notably, mitochondria not only produce most of a cell’s ROS, but they are also more susceptible than the nucleus to their own deleterious effects, due to the short half-lives of ROS in cells (≤1 μs) which limit their diffusion [38]. Since the respiratory chain located in IMM is the main site for generating ROS, high levels of oxidative stress in cells may be caused by mitochondria targeting drugs, causing oxidative damage to cellular components and leading to cell death [39].

Given the increased metabolic activity of cancer cells, mitochondrial hyperactivity in malignant transformation makes them preferential targets in anticancer therapy. Because of the increased production of ROS and the absence of robust protective mechanisms, cancer cells’ mitochondria result particularly susceptible to oxidative stress from other external factors such as IR [40]. In addition, leakage of the exceeding ROS from mitochondria induces oxidative stress affecting both n- and mtDNA and other cellular constituents [34]. As a result, therapeutic IR doses are able to increase mitochondrial oxidative stress, affecting their bioenergetic and biosynthetic metabolism and, in turn, inducing programmed cell death.

## 3. Effect of Radiation Quality on ROS Generation

It should be noted that the amount of ROS produced by a cell depends on the radiation type, and compared with low-LET and high-LET particles, e.g., carbon ions or protons toward the distal edge of the spread-out Bragg peak (SOBP), are much more effective in triggering diverse biological effects in mammalian cells, including genomic instability and malignant transformation [41]. This phenomenon is well-described by the relative biological effectiveness (RBE) parameter, which for instance for protons increases along with LET at the end of the SOBP [42]. A possible explanation for the higher efficiency of high LET radiations in increasing cellular ROS levels could be a stronger impairment of the antioxidative capacities of the exposed cells, as recently observed in human fibroblasts exposed to photons and carbon ions [43].

Regarding high-LET exposures, their specificity appears to rely on two main factors, namely the precise targeting of tumors associated with a high local energy deposition and the ability to induce mitochondrial dysfunction associated with non-irreversible apoptosis. Both factors contribute to the higher RBE typical for high-LET radiation. It is thought that the destructive power of highly dense ionization tracks causes mitochondrial dysfunction, metabolic distress, and widespread ROS that can overwhelm cancer cell defenses. Such an efficiency could be explained by the fact that particles could trigger an enhanced bystander effect since mitochondria are the main contributors to the regulation of innate and adaptive immunity, playing a crucial role in immunogenic antitumor response [44,45,46]. Indeed, the activation of neighboring cells and of circulating active immune cells enables more efficiently recognizing and eliminating aberrant cancer clones, in turn affecting tumor growth and metastasis formation. To support this hypothesis, several studies on patients with radioresistant solid tumors reported that particle therapy (in particular with carbon ions) is more beneficial in long-term outcomes with fewer side effects or fewer metastases and secondary cancers [47,48]. Interestingly, very recent data reveal that somewhat similar results may be obtained with conventional photon therapy in combination with DNA repair and immune checkpoint inhibitors [49,50,51]. On this basis, it could result in great interest to further explore the effect of radiation-induced mitochondrial dysfunction by employing low- and high-LET radiations.

## 4. Radioresistance and Mitochondria: Roles of Hypoxia and Metabolic Alterations

Radioresistance represents the main cause of RT treatment failure, ultimately leading to recurrence and metastatic progression. Although the mechanism underlying the development of radioresistance is fogged by a number of cellular signaling pathways and factors that contribute to such a complex process [52], an increasing number of studies demonstrates its close relation to alterations in tumor metabolism [53,54]. The development of tumor hypoxia and the associated metabolic pathways are one of the most important contributors [55], and, from a clinical standpoint, the main cause of cellular radioresistance is conferred by glycolytic/mitochondrial metabolic changes [56]. The decrease in oxygen availability means that cells must adapt their metabolic program to maintain the catabolic and anabolic reactions that rely on the availability of ATP normally supplied by OXPHOS. In this context, the metabolic reprogramming under hypoxia is mainly dependent on hypoxia-inducible factor 1-alpha (HIF-1α) transcription factor activity [57,58]. In general, HIF-1α signaling supports anaerobic ATP production and downregulates OXPHOS, thus reducing the cell’s reliance on oxygen-dependent energy production [59]. Since mitochondria are fundamental for oxygen-dependent metabolism, HIF-1α-dependent adaptation to hypoxia affects mitochondrial functions at many levels [60].

As a consequence of oxidative metabolism, which occurs in tumor cells, elevated amounts of ROS are produced from the mitochondrial ETC. High levels of mitochondrial ROS in turn activate signaling pathways proximal to the mitochondria to promote tumor cell proliferation and tumorigenesis [61]. However, if ROS are allowed to accumulate, cells undergo apoptosis [62]. Therefore, cancer cells generate an abundance of nicotinamide adenine dinucleotide phosphate (NADPH) in the mitochondria and cytosol to support high antioxidant activity and prevent the accumulation of potentially harmful ROS [63,64]. Thus, both glucose-dependent metabolic pathways and mitochondrial metabolism are essential for cancer cell proliferation.

The high rate of glycolysis in tumor cells is a consequence of deregulated signaling pathways, such as the phosphatidylinositol 3-kinase (PI3K) pathway and activation of oncogenes such as MYC and KRAS. In turn, this allows the generation of glycolytic intermediates that can funnel into multiple subsidiary biosynthetic pathways necessary for cell proliferation, such as the pentose phosphate pathway (PPP) for NADPH and nucleotide production [65]. The majority of cancer cells are known to produce energy primarily through accelerated glycolysis, followed by lactic acid fermentation even under normoxic conditions. This metabolic phenomenon, known as the Warburg effect, results less efficient, in terms of the amount of ATP for molecules of glucose produced, when compared with mitochondrial OXPHOS. However, the PPP-accompanying pathway can favor cancer cells by producing numerous substrates required for malignant proliferation and radioresistance [66]. Because many malignant cells are found themselves under hypoxic conditions during tumor growth, the metabolic reprogramming from OXPHOS to accelerated glycolysis is a key aspect of cancer cells’ adaptive response to hypoxia. In addition, under hypoxic circumstances, DNA free radicals can be reduced to their original form, decreasing ROS production and weakening radiation-induced DNA damage [67]. Hence, damage to cancer-cells DNA is greatly reduced at low oxygen levels, especially with low-LET sparse IR (e.g., photons) [68], since the influence of oxygen pressure increases as LET decreases, resulting in a condition called hypoxia-induced radioresistance, a common feature of solid tumors [69]. The influence of oxygen is well-defined by the OER, which compares the ratio of doses in hypoxic and normoxic conditions to obtain the same endpoint from a biological point of view, with values of ~2.5–3 for photons and ~1 (no oxygen effect) for high-LET radiations [70].

Hypoxia generally presents as a consequence of the rapid proliferation of malignant cells that exceeds their blood supply, therefore diminishing nutrients and oxygen available for the cells [71]. Hypoxic tumors have been reported to be highly aggressive, resistant to common strategies such as chemotherapy and RT, and associated with poor prognosis [72]. In fact, as stated above, the hypoxic microenvironment represents a significant barrier and affects the clinical outcome of RT requiring a higher radiation dose (up to three times the normal radiation) to achieve the desired apoptotic effect with respect to normoxic malignancies [73]. In this context, several efforts to improve clinical response by targeting cellular glucose and mitochondrial metabolism have been attempted [54,74]. In 2014, Shimura et al. reported how radioresistance is influenced by serine/threonine kinase (AKT)-mediated enhanced aerobic glycolysis acquired by tumor cells [54], demonstrating that radioresistant cells have higher lactate production rates and enhanced aerobic glycolysis compared with parental cells, thus suggesting that tumor cell metabolic pathway, in which mitochondria play a key role, is an attractive target to eliminate radioresistant clones and improve RT efficacy. On the other hand, Bol and colleagues investigated the impact of inhibition of the mitochondrial oxygen uptake on the tumor sensitivity to RT [74]. Their results underlined that even a subtle change in oxygen availability, due to cellular oxygen consumption, could modulate the response of malignant clones to radiation. These results provide a relevant rationale for combining therapeutic interventions aimed at decreasing the oxygen consumption rate of tumor cells during RT, resulting in anti-metabolic approaches.

## 5. Mitochondria and Epigenetics

In radiation oncology, past research has mainly focused on the direct damaging effects of IR on the DNA. However, chronic disruptions in mitochondrial metabolism and other redox-sensitive pathways, initiated during irradiation, also provide fundamental changes in the intracellular signaling environment to elevate oxidative injury and alter cellular physiology, prompting genomic instability [22,75].

In this regard, mitochondria play a major role in the radiation-induced genomic instability through epigenetic mechanisms.

As widely known and reported elsewhere, epigenetics is the study of heritable phenotype alterations not related to a change in DNA sequence [76]. Its regulation is largely reported as an important biological process involved in cancer development and spread, enabling adaptation to the microenvironment and growth advantage for tumor clones over normal cells [77]. Epigenetic modifications in nDNA have been well described and characterized and comprise different layers of regulation, including covalent modifications of DNA bases, post-translational histones modifications, and RNA and non-coding RNA (ncRNA) modulation [78,79,80,81]. Similar to its nuclear counterpart, mtDNA is under epigenetic regulation, the so-called mitoepigenetics [82,83]; mtDNA is mostly hypomethylated with respect to the nDNA and shows a slightly different epigenetic regulation, mainly related to the methylation activity [84] of the mitochondria-specific DNA methyltransferase (mtDNMT1) [85]. In particular, RT-derived oxidative stress deeply affects mtDNA methylation, by impairing methylation sites availability (by oxidizing CpG islets), as well as inhibiting mtDNMT1 activity, in turn altering mtDNA transcription and mitochondrial functions.

While the above-mentioned relationship among persistent oxidative stress, epigenetics, and mitochondrial function describes how RT acts on mtDNA, arguably it is also important to consider a further perspective, in which the RT-induced redox perturbations of mitochondrial functions represent the driving force affecting the whole epigenetic machinery, and in turn the regulation of gene expression, and finally genome integrity [22,86,87]. Accordingly, the physiologic mitochondrial activity involves the production of several cofactors crucial for epigenetic marks (e.g., ATP and acetyl-CoA) [82,88] and for the functioning of the whole epigenetic machinery [10,86,89]. Overall, as reported in a recent review by Baulch and colleagues, metabolic activities in the mitochondria are essential to provide the ATP required for phosphorylation and the acetyl coenzyme A (acetyl-CoA) needed for acetylation of histone tails [88]. Therefore, RT-derived redox perturbation of normal mitochondria functioning can, thus, affect the whole gene expression through different and yet-tobe unraveled layers of regulation [78,79,80,81].

## 6. Mitochondria-Targeting Strategies to Improve RT Effects

Due to their crucial role, mitochondria-targeting strategies in tumor cells have gained increasing attention. For example, the anticancer agent Lonidamine (LND) has been shown to selectively inhibit aerobic glycolysis in cancer cells and succinate:ubiquinone reductase activity of complex II, resulting in increased ROS [90,91]. The effectiveness of LND in combination with RT for the treatment of breast, brain, melanoma, prostate, and ovarian tumors has already been tested [92,93,94]. Modifying chemotherapy/RT drugs with mitochondria targeting units, such as LND, showed promising results also in radio-resistant malignant melanoma cells [95], although additional information is needed to clarify LND/RT therapeutic effectiveness before reaching the clinical side. Moreover, increasing oxygen delivery to counteract hypoxic radioresistance (e.g., hyperbaric oxygen) has been intensively explored; on the other hand, reduction in oxygen demand has attracted considerable attention, particularly with clinically relevant agents that are reported to overcome hypoxic radioresistance [96,97]. For example, nonsteroidal anti-inflammatory drugs (NSAIDs), such as piroxicam, indomethacin, and diclofenac, were demonstrated to increase tumor oxygenation when tested in murine transplantable liver tumor (TLT) and fibrosarcomas by influencing mitochondrial respiration [98]. When irradiation was applied at the time of maximal reoxygenation, the tumor radiosensitivity was enhanced (regrowth delay increased by a factor of 1.7), equivalent to the radiosensitization effect generated by hyperoxic gas breathing [55]. These results showed the potential utility of an acute administration of NSAIDs for radiosensitizing tumors, providing a new potential rationale for the treatment schedule when combining NSAIDs and radiotherapy. Such an effect triggered by anti-inflammatory agents has been also confirmed with steroid agents such as glucocorticoids, shown to promote tumor oxygenation by lowering oxygen consumption, thus resulting in an increase in tumor radiosensitivity [99].

Glucocorticoids such as hydrocortisone, dexamethasone, and prednisolone were tested, and when irradiation was applied, the tumor radiosensitivity was enhanced as observed with NSAIDs.

Metformin, a commonly used anti-diabetes drug, improves tumor oxygenation by inhibiting mitochondrial complex I [96], and the combination of radiation and metformin is being studied in various clinical trials [100]. In a recent review, Rao et al. [101] analyzed 17 studies on metformin-enhanced RT in patients with diabetes and different sites of tumor, showing that metformin correlated with improved tumor response to treatment, thus suggesting that it might represent an effective and inexpensive means to improve RT outcome with an optimal therapeutic ratio. Similar findings on the beneficial effect of metformin in RT were reported by Zannella and colleagues [96]. In this retrospective analysis, the authors found that metformin use was associated with a significant decrease in early biochemical relapse rates in 504 patients with localized prostate cancer under RT. In addition, Atovaquone, an anti-malarian drug and mitochondrial complex III-inhibitor, showed promising activity, reducing oxygen consumption by more than 80% in a variety of cancer cell lines and causing a delay in tumor growth [102]. The potential of this drug in combination with RT is under investigation in the ARCADIAN trial at Oxford University, with the aim to assess its safety and treatment improvements in the survival of patients with non-small cell lung cancer. Finally, Auranofin, an anti-arthritis drug considered for combined chemotherapy due to its ability to impair the redox homeostasis in tumor cells, has shown to significantly improve tumor radioresponse, when combined with buthionine sulfoximine, by arresting oxygen consumption in mitochondria [103]. A summary of the above-mentioned mitochondria-targeting strategies is provided in Table 1.

Multiple other modalities involving the modification of already available strategies with mitochondria targeting units to induce ROS formation, including photodynamic therapy (PDT) and photothermal therapy (PTT), have been also investigated, resulting promising in certain settings.

More in depth, PDT represents a clinically approved therapeutic procedure which uses three essential components—a special photosensitizer drug, light, and oxygen—to kill cancer and other abnormal cells [104]. The therapy consists in the administration of a photosensitizing agent followed by irradiation of a specific wavelength: the absorption of light by the photosensitizing drug results in the transfer of energy to molecular oxygen. This leads to the formation of ROS, O_2_^−^⋅ (type I reaction), or O_2_ (type II reaction) only in the light-exposed region [105], which destroys the cells in which they have developed, leading to direct tumor cell death, damage to the microvasculature, and induction of a local inflammatory reaction.

Since ROS have a short half-life (40 ns) and diffusion ratio (<20 nm), the best effect is achieved when PS is transported to mitochondria for in situ ROS generation, enhancing the efficacy of PDT [106]. In particular, to increase the mitochondrial uptake in PDT, several lipophilic and cationic groups are used to penetrate the negatively charged mitochondrial membrane, such as organic phosphine/sulfur salt (e.g., triphenylphosphonium (TPP)), QA salts (rhodamine and rhodamine derivatives and pyridinium) transition metal complexes, guanidinium, and bisguanidinium [39].

TPP is also used in PTT, where it is the most common mitochondria-targeting unit connected to photothermal agents. PTT, an efficient complement to standard cancer treatments as RT and chemotherapy, relies on activation of PS by pulsed laser irradiation to generate heat for thermal ablation of cancer by inducing apoptosis in tumor tissues, and its advantages include deep penetration and minimal effects on the surrounding healthy tissues [107].

In a study published in 2015, Jung and colleagues [108] designed and synthesized a specific mitochondrion-targeting compound that was then tested according to its ability to induce significant cell hyperthermia upon near-infrared (NIR) irradiation. The compound was able to enhance the temperature of NIR-irradiated cells by more than 2 °C, boosting the therapeutic efficacy of hyperthermia treatment. A subsequent publication by the same group [109] described another TPP-based mitochondrion-targeting compound which showed the capability to further increase the temperature up to 13.5 °C, therefore confirming the potential of mitochondria-targeting modification and the subsequent ROS production and representing a new generation of PTT system.

Overall, the above-described techniques hold the promise to overcome hypoxia-induced radioresistance, and their combination with RT may open new avenues for novel therapeutic approaches [39,110,111].

**Table 1:** Summary of the combined principal radiotherapy−mitochondria targeting strategies to enhance radiosensitivity.

Compound Name	Mitochondria-Targeted Unit	Testing Models	References
***Lonidamine***	complex II	human glioma cell lines	Prabhakara et al. [92], 2018
		human glioma cell lines	Kalia, V et al. [93], 2009
		non-small cell lung cancer cell lines	Meijer T W H et al. [94], 2018
***Non-steroidal anti-inflammatory drugs***	complex I	murine TLT liver tumors and FSaII fibrosarcomas	Crokart et al. [98], 2005
***Glucocorticoids***	complex I and complex III	murine TLT liver tumors and FSaII fibrosarcomas	Crokart et al. [99], 2007
***Metformin***	complex I	prostate cancer	Zannella VE et al. [96], 2013
		prostate cancer	Taira AV et al. [112], 2014
		rectal cancer	Skinner HD et al. [113], 2013
		esophageal cancer	Spierings et al. [114], 2015;
		liver cancer	Jang et al. [115], 2015
		head and neck cancer	Spratt et al. [116], 2016
***Atovaquone***	complex III	FaDU and HCT116 xenografts in nude mice	Ashton et al. [102], 2016
		non-small cell lung cancer	ARCADIAN TRIAL (currently in recruiting phase)
***Auranofin***	mitochondrial thioredoxin reductase	H1299 tumor cell lines xenografted in murine models	Wang et al. [103], 2017

## 7. Conclusions and Future Perspectives

It is still a matter of debate how mitochondrial dysfunction contributes to radiation-derived genetic instability through epigenetic changes, metabolic reprogramming, and/or ROS generation. Unfortunately, partly due to differences in experimental design (e.g., dosage or quality of radiation and types of the tissue or model system), there is a lack of clarity about the precise role of the above-mentioned points, and the present literature review might provide many worthwhile observations to be further explored.

As of today, mitochondria have attracted considerable attention as targets for the development of novel anticancer agents, essentially for their central role in the pathways regulating cell death and chemio- and radio-resistance of cancer cells. Overall, a better understanding of mitochondria-dependent mechanisms of cancer cell resistance, also expanding the current knowledge on mitochondrial epigenetics, would lead to the development of more effective therapeutic strategies, allowing the design of combination therapies using mitochondria-targeting agents in association with current therapeutic regimens. Notably, the role of mitochondria in radiation-induced DNA instability via epigenetic mechanisms is a new subject with potentially significant and far-reaching implications. Arguably, studies supporting a direct link among IR exposure, genomic instability, and mitochondrial dysfunction are still lacking, and future efforts are warranted for unifying such three components.

Finally, further insights into the context of hypoxia and ROS generation could be derived from the use of protons and hadrons, which bring a smaller OER and a greater RBE as compared with photons, and from the analysis of their interaction with mitochondria. Refined strategies for modulating mitochondrial functions in selected tumor types are warranted to fully exploit the therapeutic potential of mitochondria-targeting drugs, with the final goal of enhancing RT effects or tackling radioresistance.
